# Crystal structure of ethyl (2*S*,2′*R*)-1′-benzyl-3-oxo-3*H*-di­spiro­[1-benzo­thio­phene-2,3′-pyrrolidine-2′,11′′-indeno[1,2-*b*]quinoxaline]-4′-carboxyl­ate

**DOI:** 10.1107/S2056989015003187

**Published:** 2015-02-21

**Authors:** J. Govindaraj, R. Raja, M. Suresh, R. Raghunathan, A. SubbiahPandi

**Affiliations:** a Department of Physics, Pachaiyappa’s College for Men, Kanchipuram 631 501, India; b Department of Physics, Presidency College (Autonomous), Chennai 600 005, India; c Department of Organic Chemistry, University of Madras, Guindy Campus, Chennai 602 025, India

**Keywords:** crystal structure, di­spiro compounds, ester, hydrogen bonding, benzo­thio­phene, pyrrolidine, indeno­[1,2-*b*]quinoxaline, biological activity, π–π inter­actions

## Abstract

In the title compound, C_35_H_27_N_3_O_3_S, the spiro-linked five-membered rings both adopt twisted conformations. The pyrrolidine ring makes dihedral angles of 80.5 (1) and 77.4 (9)° with the benzo­thio­phene ring system and the quinoxaline ring system, respectively. The S atom and C=O unit of the benzo­thio­phene ring system are disordered over two opposite orientations in a 0.768 (4):0.232 (4) ratio. The atoms of the ethyl side chain are disordered over two sets of sites in a 0.680 (16):0.320 (16) ratio. In the crystal, mol­ecules are linked by C—H⋯O, C—H⋯N and π–π inter­actions [shortest centroid–centroid distance = 3.4145 (19) Å], resulting in a three-dimensional network.

## Related literature   

For general background to spiro compounds and their biological activity, see: Pradhan *et al.* (2006 ▸); Saeedi *et al.* (2010 ▸); Dandia *et al.* (2011 ▸); He *et al.* (2003 ▸). For uses of pyrrolidine and quinoxaline derivatives, see: Raj *et al.* (2003 ▸); Zarranz *et al.* (2003 ▸). For a related structure, see: Kannan *et al.* (2013 ▸).
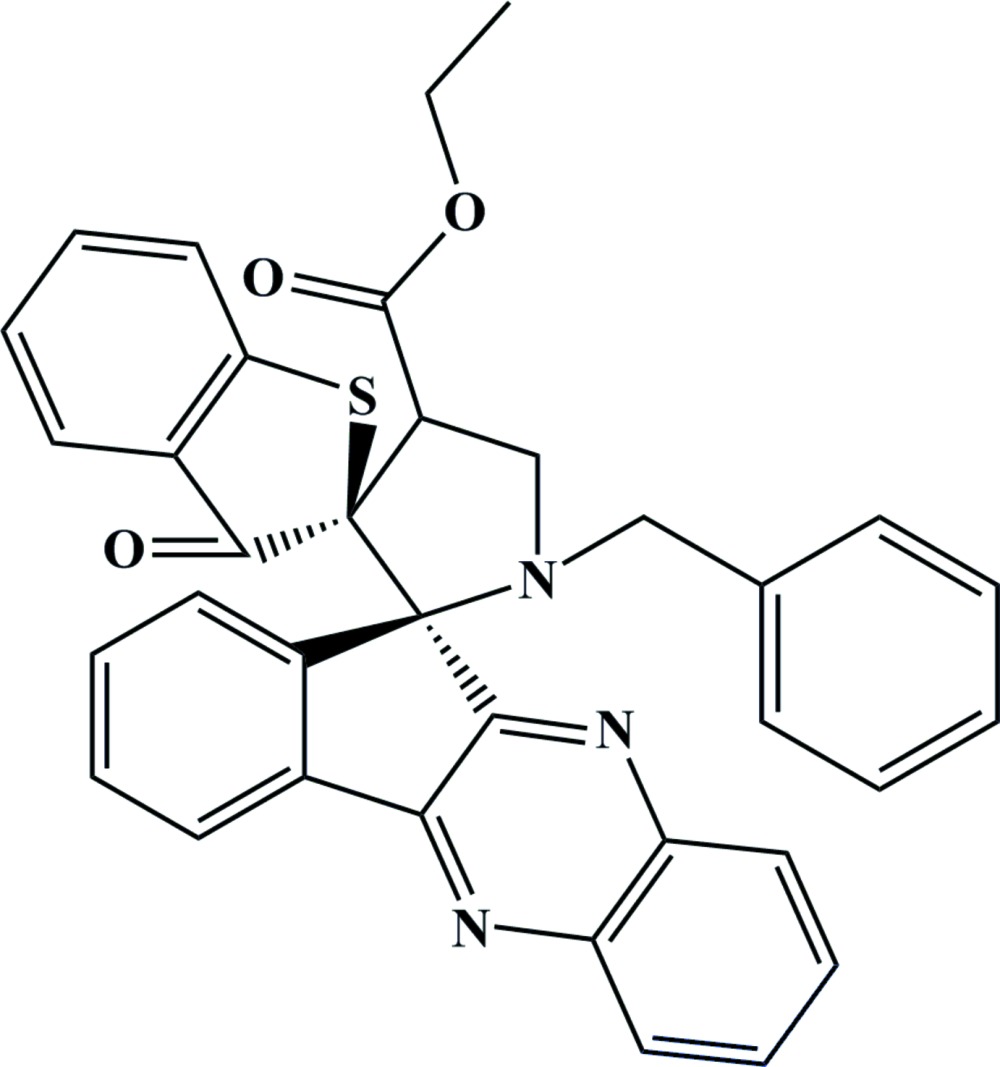


## Experimental   

### Crystal data  

C_35_H_27_N_3_O_3_S
*M* *_r_* = 569.66
Monoclinic, 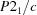

*a* = 11.3893 (5) Å
*b* = 15.1181 (7) Å
*c* = 16.7136 (7) Å
β = 100.766 (2)°
*V* = 2827.2 (2) Å^3^
*Z* = 4
Mo *K*α radiation
μ = 0.16 mm^−1^
*T* = 293 K
0.24 × 0.20 × 0.19 mm

### Data collection  

Bruker SMART APEXII CCD diffractometer
Absorption correction: multi-scan (*SADABS*; Bruker, 2008 ▸) *T*_min_ = 0.963, *T*_max_ = 0.971
26003 measured reflections
7078 independent reflections
4934 reflections with *I* > 2σ(*I*)
*R*_int_ = 0.034

### Refinement  

*R*[*F* ^2^ > 2σ(*F* ^2^)] = 0.047
*wR*(*F* ^2^) = 0.128
*S* = 1.07
7078 reflections
440 parameters
82 restraints
H atoms treated by a mixture of independent and constrained refinement
Δρ_max_ = 0.21 e Å^−3^
Δρ_min_ = −0.18 e Å^−3^

### 

Data collection: *APEX2* (Bruker, 2008 ▸); cell refinement: *SAINT* (Bruker, 2008 ▸); data reduction: *SAINT*; program(s) used to solve structure: *SHELXS97* (Sheldrick, 2008 ▸); program(s) used to refine structure: *SHELXL97* (Sheldrick, 2008 ▸); molecular graphics: *ORTEP-3 for Windows* (Farrugia, 2012 ▸); software used to prepare material for publication: *SHELXL97* and *PLATON* (Spek, 2009 ▸).

## Supplementary Material

Crystal structure: contains datablock(s) global, I. DOI: 10.1107/S2056989015003187/hb7364sup1.cif

Structure factors: contains datablock(s) I. DOI: 10.1107/S2056989015003187/hb7364Isup2.hkl

**Fig. 1.**
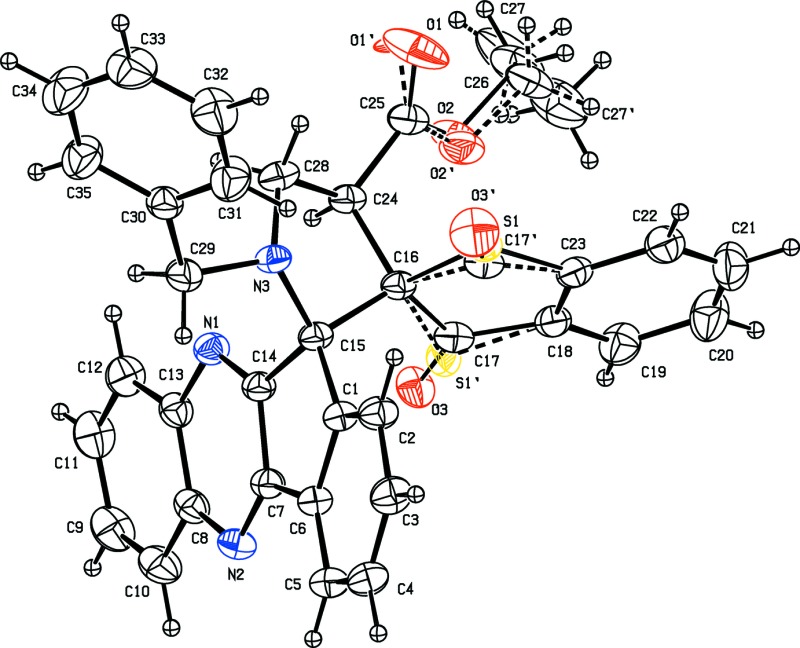
The molecular structure showing displacement ellipsoids drawn at the 30% probability level.

. DOI: 10.1107/S2056989015003187/hb7364fig1.tif
The mol­ecular structure showing displacement ellipsoids drawn at the 30% probability level.

**Fig. 2.**
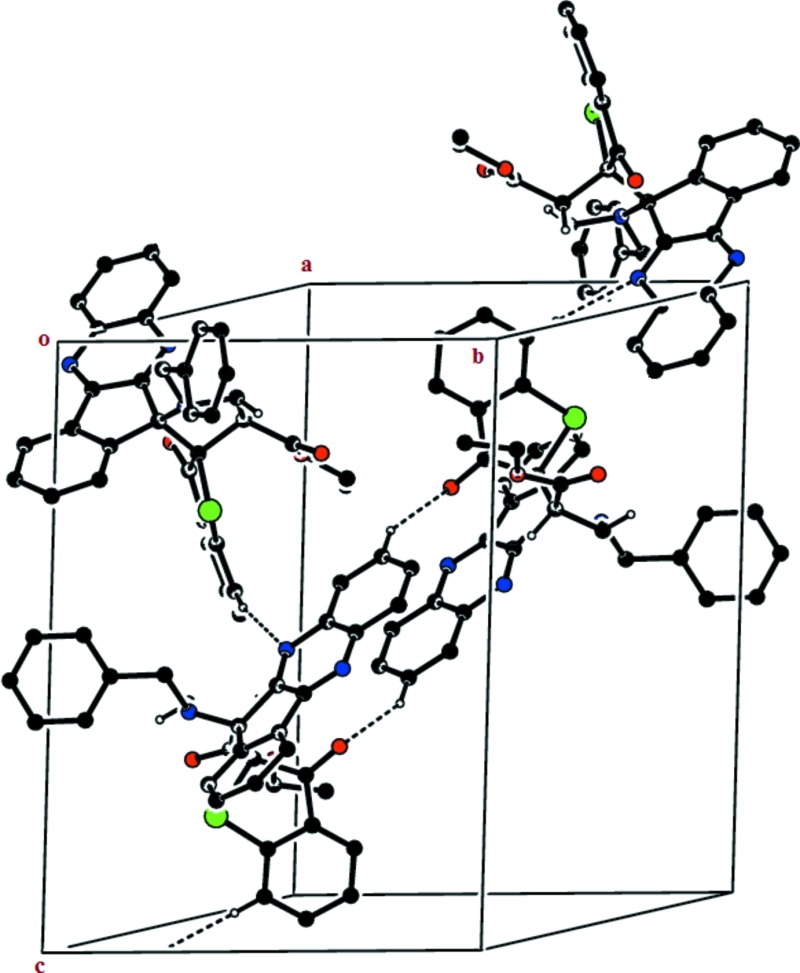
The packing of the title compound, viewed along the C-axis.

. DOI: 10.1107/S2056989015003187/hb7364fig2.tif
The packing of the title compound, viewed along the C-axis.

CCDC reference: 1049572

Additional supporting information:  crystallographic information; 3D view; checkCIF report

## Figures and Tables

**Table 1:** Hydrogen-bond geometry (, )

*D*H*A*	*D*H	H*A*	*D* *A*	*D*H*A*
C11H11O3^i^	0.93	2.38	3.222(3)	150
C22H22N1^ii^	0.93	2.61	3.523(3)	167

Symmetry codes: (i) 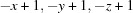; (ii) 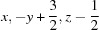.

## supplementary crystallographic information

### S1. Comment

Spiro compounds have received considerable interest due to their biological properties (Pradhan *et al.*, 2006). Thus, further spiroheterocycle compounds have been prepared and characterized (Saeedi *et al.*, 2010); Dandia *et al.*, 2011). In addition, quinoxaline derivatives also showed various biological activites (He *et al.*, 2003). Pyrrolidine derivatives are found to have anticonvulsant, antimicrobial and antifungal activities against various pathogens (Amal Raj *et al.*, 2003). Quinoxaline derivatives shown antibacterial, antiviral and anticancer properties (Zarranz *et al.*, 2003).

The X-ray study confirmed the molecular structure and atomic connectivity for the title compound, as illustrated in Fig.1. The pyrrolidine and cyclopentane rings adopts twisted conformation, with puckering parameters q_2_=0.4633 (18), φ_2_ = 231.6 (1) and q_2_ = 0.1013, φ_2_ = 302.3 (10). The five membered ring tetrahydrothiophene ring adopts envelope conformation, with the lowest asymmetry parameters ΔCS(S1—C23) = 1.6 (3)°. The pyrrolidine ring makes dihedral angles of 80.5 (1) and 77.36 (9)° with the benzothiophene ring system and the quinoxaline rings. The pyrrolidine ring, the largest deviation from the mean plane -1.1084 and -0.4921 Å for the C27 and C27' atom.

In the crystal, the molecules are linked by C—H···O, C—H···N and π–π interactions [centroid-centroid distance = 3.4146 Å].

### S2. Experimental

A mixture of ninhydrin(1 mmol) and 1,2-phenylenediamine(1 mmol) was stirred for 10 min in 10 ml of methanol followed by addition of *N*-Benzyl glycine(1 mmol). To this mixture, a solution of (*E*)-ethyl 2-(3-oxobenzo[*b*] thiophen-2(3*H*)-ylidene)acetate (1.0 mmol) in 10 ml of methanol was added. The mixture was then refluxed until completion of the reaction as evidenced by TLC. The solvent was removed under reduced pressure and the crude product obtained was purified by column chromatography using petroleum ether/ ethylacetate(4:1) as eluent. The product was dissolved in ethyl acetate and heated for two minutes. The resulting solution was subjected to crystallization by slow evaporation of the solvent for 48 h resulting in colourless blocks.

### S3. Refinement

N and C-bound H atoms were positioned geometrically (C–H = 0.93–0.98 Å) and allowed to ride on their parent atoms, with *U*_iso_(H) = 1.5*U*_eq_(C) for methyl H atoms and 1.2*U*_eq_(C) for all other H atoms.

### Crystal data

**Table:**

C_35_H_27_N_3_O_3_S	*Z* = 4
*M**_r_* = 569.66	*F*(000) = 1192
Monoclinic, *P*2_1_/*c*	*D*_x_ = 1.338 Mg m^−^^3^
Hall symbol: -P 2ybc	Mo *K*α radiation, λ = 0.71073 Å
*a* = 11.3893 (5) Å	Cell parameters from 7078 reflections
*b* = 15.1181 (7) Å	µ = 0.16 mm^−^^1^
*c* = 16.7136 (7) Å	*T* = 293 K
β = 100.766 (2)°	Block, colourless
*V* = 2827.2 (2) Å^3^	0.24 × 0.20 × 0.19 mm

### Data collection

**Table:**

Bruker SMART APEXII CCD diffractometer	7078 independent reflections
Radiation source: fine-focus sealed tube	4934 reflections with *I* > 2σ(*I*)
Graphite monochromator	*R*_int_ = 0.034
ω and φ scans	θ_max_ = 28.7°, θ_min_ = 1.8°
Absorption correction: multi-scan (*SADABS*; Bruker, 2008)	*h* = −14→15
*T*_min_ = 0.963, *T*_max_ = 0.971	*k* = −20→15
26003 measured reflections	*l* = −22→22

### Refinement

**Table:**

Refinement on *F*^2^	Primary atom site location: structure-invariant direct methods
Least-squares matrix: full	Secondary atom site location: difference Fourier map
*R*[*F*^2^ > 2σ(*F*^2^)] = 0.047	Hydrogen site location: inferred from neighbouring sites
*wR*(*F*^2^) = 0.128	H atoms treated by a mixture of independent and constrained refinement
*S* = 1.07	*w* = 1/[σ^2^(*F*_o_^2^) + (0.0446*P*)^2^ + 0.8872*P*] where *P* = (*F*_o_^2^ + 2*F*_c_^2^)/3
7078 reflections	(Δ/σ)_max_ = 0.001
440 parameters	Δρ_max_ = 0.21 e Å^−^^3^
82 restraints	Δρ_min_ = −0.18 e Å^−^^3^

### Special details

**Table:**

Geometry. All e.s.d.'s (except the e.s.d. in the dihedral angle between two l.s. planes) are estimated using the full covariance matrix. The cell e.s.d.'s are taken into account individually in the estimation of e.s.d.'s in distances, angles and torsion angles; correlations between e.s.d.'s in cell parameters are only used when they are defined by crystal symmetry. An approximate (isotropic) treatment of cell e.s.d.'s is used for estimating e.s.d.'s involving l.s. planes.
Refinement. Refinement of *F*^2^ against ALL reflections. The weighted *R*-factor *wR* and goodness of fit *S* are based on *F*^2^, conventional *R*-factors *R* are based on *F*, with *F* set to zero for negative *F*^2^. The threshold expression of *F*^2^ > σ(*F*^2^) is used only for calculating *R*-factors(gt) *etc*. and is not relevant to the choice of reflections for refinement. *R*-factors based on *F*^2^ are statistically about twice as large as those based on *F*, and *R*- factors based on ALL data will be even larger.

### Fractional atomic coordinates and isotropic or equivalent isotropic displacement parameters (Å^2^)

**Table:**

	*x*	*y*	*z*	*U*_iso_*/*U*_eq_	Occ. (<1)
C1	0.94305 (15)	0.59196 (11)	0.32387 (10)	0.0390 (4)	
C2	1.02494 (16)	0.61651 (12)	0.27599 (11)	0.0474 (4)	
H2	1.0270	0.6742	0.2569	0.057*	
C3	1.10351 (18)	0.55315 (14)	0.25723 (12)	0.0536 (5)	
H3	1.1592	0.5689	0.2255	0.064*	
C4	1.10079 (19)	0.46711 (14)	0.28464 (12)	0.0557 (5)	
H4	1.1534	0.4255	0.2703	0.067*	
C5	1.02072 (18)	0.44219 (12)	0.33317 (12)	0.0504 (4)	
H5	1.0191	0.3844	0.3520	0.060*	
C6	0.94291 (15)	0.50536 (11)	0.35315 (10)	0.0400 (4)	
C7	0.85854 (15)	0.50023 (11)	0.40909 (10)	0.0387 (4)	
C8	0.74977 (16)	0.44736 (12)	0.49827 (11)	0.0441 (4)	
C9	0.70512 (19)	0.37615 (14)	0.53845 (13)	0.0586 (5)	
H9	0.7266	0.3184	0.5285	0.070*	
C10	0.6306 (2)	0.39205 (16)	0.59184 (14)	0.0643 (6)	
H10	0.6010	0.3448	0.6177	0.077*	
C11	0.59810 (19)	0.47810 (16)	0.60827 (13)	0.0627 (6)	
H11	0.5490	0.4878	0.6461	0.075*	
C12	0.63773 (19)	0.54804 (14)	0.56935 (12)	0.0559 (5)	
H12	0.6151	0.6052	0.5804	0.067*	
C13	0.71247 (16)	0.53441 (12)	0.51270 (10)	0.0427 (4)	
C14	0.81360 (15)	0.58677 (10)	0.41819 (10)	0.0378 (4)	
C15	0.85285 (15)	0.64971 (10)	0.35577 (10)	0.0371 (4)	
C16	0.74095 (15)	0.67850 (10)	0.29184 (10)	0.0369 (4)	
C18	0.60427 (17)	0.61769 (11)	0.16969 (10)	0.0455 (4)	
C19	0.5082 (2)	0.57525 (14)	0.12280 (13)	0.0624 (6)	
H19	0.4675	0.5308	0.1449	0.075*	
C20	0.4738 (2)	0.60023 (18)	0.04237 (14)	0.0769 (7)	
H20	0.4089	0.5727	0.0098	0.092*	
C21	0.5351 (2)	0.66581 (17)	0.01002 (13)	0.0723 (7)	
H21	0.5104	0.6822	−0.0441	0.087*	
C22	0.6315 (2)	0.70724 (14)	0.05597 (12)	0.0562 (5)	
H22	0.6731	0.7507	0.0334	0.067*	
C23	0.66571 (16)	0.68277 (11)	0.13706 (10)	0.0440 (4)	
C24	0.68928 (15)	0.75054 (10)	0.34217 (10)	0.0399 (4)	
H24	0.6505	0.7203	0.3821	0.048*	
C25	0.59899 (18)	0.81252 (12)	0.29378 (12)	0.0486 (4)	
C28	0.80035 (16)	0.79654 (11)	0.38832 (12)	0.0469 (4)	
H28A	0.8121	0.8530	0.3633	0.056*	
H28B	0.7934	0.8066	0.4446	0.056*	
C29	0.99701 (19)	0.73891 (12)	0.45364 (12)	0.0520 (5)	
H29A	1.0523	0.6910	0.4498	0.062*	
H29B	0.9653	0.7307	0.5031	0.062*	
C30	1.06247 (16)	0.82614 (12)	0.45754 (11)	0.0473 (4)	
C31	1.0953 (2)	0.86170 (15)	0.38916 (14)	0.0678 (6)	
H31	1.0783	0.8308	0.3402	0.081*	
C32	1.1527 (2)	0.94207 (17)	0.39172 (16)	0.0754 (7)	
H32	1.1738	0.9649	0.3447	0.091*	
C33	1.1787 (2)	0.98798 (16)	0.46243 (18)	0.0767 (7)	
H33	1.2177	1.0422	0.4643	0.092*	
C34	1.1471 (3)	0.95415 (17)	0.53055 (17)	0.0869 (8)	
H34	1.1647	0.9853	0.5793	0.104*	
C35	1.0890 (2)	0.87391 (15)	0.52796 (14)	0.0687 (6)	
H35	1.0674	0.8519	0.5751	0.082*	
N1	0.74601 (13)	0.60603 (9)	0.47047 (8)	0.0428 (3)	
N2	0.82817 (14)	0.43036 (9)	0.44648 (9)	0.0460 (4)	
N3	0.89985 (13)	0.73649 (9)	0.38373 (9)	0.0399 (3)	
S1	0.78568 (8)	0.73399 (5)	0.20235 (4)	0.0406 (3)	0.768 (4)
C17	0.6529 (4)	0.6080 (3)	0.2563 (3)	0.0435 (10)	0.768 (4)
O3	0.6242 (2)	0.54889 (17)	0.29998 (16)	0.0534 (6)	0.768 (4)
S1'	0.6310 (4)	0.5815 (3)	0.2707 (2)	0.0492 (13)	0.232 (4)
C17'	0.7539 (7)	0.7084 (5)	0.2082 (4)	0.0435 (10)	0.232 (4)
O3'	0.8375 (8)	0.7589 (5)	0.2028 (5)	0.075 (3)	0.232 (4)
O1	0.6076 (16)	0.8911 (4)	0.2969 (10)	0.072 (3)	0.320 (16)
O2	0.5072 (16)	0.7644 (13)	0.2591 (17)	0.067 (4)	0.320 (16)
C26	0.4105 (14)	0.8121 (16)	0.2061 (14)	0.067 (3)	0.320 (16)
H26A	0.3973	0.7864	0.1520	0.080*	0.320 (16)
H26B	0.4333	0.8735	0.2018	0.080*	0.320 (16)
C27	0.2996 (15)	0.8074 (19)	0.2390 (16)	0.133 (7)	0.320 (16)
H27A	0.2866	0.7476	0.2544	0.200*	0.320 (16)
H27B	0.2337	0.8264	0.1981	0.200*	0.320 (16)
H27C	0.3063	0.8451	0.2858	0.200*	0.320 (16)
O1'	0.6159 (7)	0.8878 (3)	0.2794 (6)	0.096 (2)	0.680 (16)
O2'	0.4957 (7)	0.7732 (6)	0.2675 (8)	0.0590 (19)	0.680 (16)
C26'	0.3996 (7)	0.8243 (8)	0.2179 (8)	0.082 (3)	0.680 (16)
H26C	0.4244	0.8455	0.1688	0.099*	0.680 (16)
H26D	0.3788	0.8747	0.2482	0.099*	0.680 (16)
C27'	0.2971 (6)	0.7641 (5)	0.1971 (6)	0.105 (3)	0.680 (16)
H27D	0.3197	0.7137	0.1684	0.158*	0.680 (16)
H27E	0.2322	0.7944	0.1632	0.158*	0.680 (16)
H27F	0.2724	0.7447	0.2461	0.158*	0.680 (16)

### Atomic displacement parameters (Å^2^)

**Table:**

	*U*^11^	*U*^22^	*U*^33^	*U*^12^	*U*^13^	*U*^23^
C1	0.0421 (9)	0.0319 (8)	0.0440 (8)	0.0016 (7)	0.0106 (7)	−0.0031 (7)
C2	0.0507 (11)	0.0400 (9)	0.0552 (10)	−0.0001 (8)	0.0195 (8)	0.0010 (8)
C3	0.0509 (11)	0.0558 (12)	0.0591 (11)	0.0034 (9)	0.0231 (9)	−0.0041 (9)
C4	0.0587 (12)	0.0512 (11)	0.0598 (11)	0.0158 (9)	0.0177 (10)	−0.0087 (9)
C5	0.0587 (12)	0.0362 (9)	0.0565 (11)	0.0100 (8)	0.0114 (9)	−0.0017 (8)
C6	0.0435 (10)	0.0322 (8)	0.0444 (8)	0.0022 (7)	0.0089 (7)	−0.0015 (7)
C7	0.0400 (9)	0.0319 (8)	0.0437 (8)	0.0011 (7)	0.0065 (7)	0.0007 (7)
C8	0.0433 (10)	0.0406 (9)	0.0469 (9)	−0.0029 (7)	0.0044 (7)	0.0082 (8)
C9	0.0612 (13)	0.0460 (11)	0.0679 (12)	−0.0066 (9)	0.0103 (10)	0.0155 (10)
C10	0.0624 (13)	0.0644 (14)	0.0676 (13)	−0.0149 (11)	0.0161 (11)	0.0222 (11)
C11	0.0571 (13)	0.0771 (16)	0.0581 (12)	−0.0105 (11)	0.0214 (10)	0.0104 (11)
C12	0.0613 (13)	0.0571 (12)	0.0530 (11)	−0.0035 (10)	0.0200 (9)	0.0040 (9)
C13	0.0440 (10)	0.0429 (9)	0.0412 (8)	−0.0031 (7)	0.0078 (7)	0.0049 (7)
C14	0.0429 (9)	0.0303 (8)	0.0407 (8)	−0.0003 (7)	0.0092 (7)	0.0011 (7)
C15	0.0430 (9)	0.0276 (7)	0.0427 (8)	−0.0009 (6)	0.0132 (7)	−0.0006 (7)
C16	0.0413 (9)	0.0288 (7)	0.0429 (8)	0.0002 (6)	0.0138 (7)	0.0020 (7)
C18	0.0559 (11)	0.0356 (9)	0.0466 (9)	0.0025 (8)	0.0141 (8)	−0.0009 (8)
C19	0.0723 (14)	0.0536 (12)	0.0625 (12)	−0.0179 (10)	0.0155 (11)	−0.0037 (10)
C20	0.0792 (17)	0.0868 (18)	0.0602 (13)	−0.0212 (14)	0.0010 (12)	−0.0115 (13)
C21	0.0876 (17)	0.0831 (17)	0.0442 (10)	−0.0041 (14)	0.0072 (11)	0.0019 (11)
C22	0.0730 (14)	0.0482 (11)	0.0522 (10)	0.0000 (10)	0.0240 (10)	0.0044 (9)
C23	0.0527 (11)	0.0344 (9)	0.0470 (9)	0.0019 (7)	0.0151 (8)	−0.0050 (7)
C24	0.0457 (10)	0.0294 (8)	0.0482 (9)	0.0019 (7)	0.0176 (7)	0.0021 (7)
C25	0.0552 (12)	0.0367 (10)	0.0581 (11)	0.0076 (8)	0.0211 (9)	0.0068 (9)
C28	0.0535 (11)	0.0306 (8)	0.0600 (10)	−0.0010 (7)	0.0195 (9)	−0.0061 (8)
C29	0.0638 (12)	0.0362 (9)	0.0516 (10)	−0.0024 (8)	−0.0003 (9)	0.0032 (8)
C30	0.0470 (10)	0.0375 (9)	0.0536 (10)	−0.0009 (8)	−0.0005 (8)	0.0009 (8)
C31	0.0799 (16)	0.0628 (14)	0.0596 (12)	−0.0206 (12)	0.0107 (11)	−0.0028 (11)
C32	0.0779 (16)	0.0680 (15)	0.0792 (15)	−0.0232 (13)	0.0114 (13)	0.0137 (13)
C33	0.0710 (16)	0.0506 (13)	0.1024 (19)	−0.0189 (11)	0.0004 (14)	−0.0009 (13)
C34	0.108 (2)	0.0666 (16)	0.0832 (17)	−0.0315 (15)	0.0093 (15)	−0.0216 (14)
C35	0.0861 (17)	0.0592 (13)	0.0603 (12)	−0.0163 (12)	0.0126 (11)	−0.0088 (11)
N1	0.0508 (9)	0.0363 (7)	0.0438 (7)	0.0002 (6)	0.0153 (6)	0.0020 (6)
N2	0.0523 (9)	0.0323 (7)	0.0536 (8)	0.0013 (6)	0.0108 (7)	0.0073 (6)
N3	0.0432 (8)	0.0269 (6)	0.0494 (8)	−0.0003 (6)	0.0082 (6)	−0.0026 (6)
S1	0.0467 (5)	0.0326 (4)	0.0454 (3)	−0.0077 (3)	0.0158 (3)	0.0044 (3)
C17	0.043 (2)	0.035 (2)	0.0557 (19)	0.0037 (14)	0.0177 (13)	0.0042 (15)
O3	0.0583 (12)	0.0485 (14)	0.0530 (13)	−0.0188 (10)	0.0092 (10)	0.0144 (10)
S1'	0.055 (2)	0.046 (3)	0.044 (2)	−0.0049 (18)	0.0047 (16)	0.0028 (17)
C17'	0.043 (2)	0.035 (2)	0.0557 (19)	0.0037 (14)	0.0177 (13)	0.0042 (15)
O3'	0.074 (6)	0.064 (5)	0.088 (5)	−0.039 (4)	0.016 (4)	0.006 (4)
O1	0.099 (8)	0.021 (4)	0.094 (5)	0.022 (4)	0.013 (4)	−0.018 (4)
O2	0.062 (6)	0.051 (6)	0.081 (6)	0.023 (4)	0.001 (5)	−0.004 (4)
C26	0.071 (7)	0.052 (6)	0.078 (6)	0.023 (5)	0.017 (5)	0.024 (5)
C27	0.074 (8)	0.160 (15)	0.169 (14)	0.047 (9)	0.032 (9)	0.097 (11)
O1'	0.066 (3)	0.054 (3)	0.163 (6)	0.001 (2)	0.009 (3)	0.054 (3)
O2'	0.049 (2)	0.036 (2)	0.088 (4)	0.005 (2)	0.003 (2)	0.015 (3)
C26'	0.054 (3)	0.070 (5)	0.115 (6)	0.018 (3)	−0.005 (3)	0.021 (3)
C27'	0.066 (3)	0.093 (4)	0.140 (6)	−0.001 (3)	−0.022 (3)	0.028 (4)

### Geometric parameters (Å, º)

**Table:**

C1—C2	1.388 (2)	C22—C23	1.389 (3)
C1—C6	1.398 (2)	C22—H22	0.9300
C1—C15	1.519 (2)	C23—C17'	1.459 (7)
C2—C3	1.386 (3)	C23—S1	1.7609 (19)
C2—H2	0.9300	C24—C25	1.510 (2)
C3—C4	1.381 (3)	C24—C28	1.521 (2)
C3—H3	0.9300	C24—H24	0.9800
C4—C5	1.381 (3)	C25—O1'	1.186 (4)
C4—H4	0.9300	C25—O1	1.193 (6)
C5—C6	1.385 (2)	C25—O2	1.316 (8)
C5—H5	0.9300	C25—O2'	1.318 (4)
C6—C7	1.463 (2)	C28—N3	1.465 (2)
C7—N2	1.306 (2)	C28—H28A	0.9700
C7—C14	1.423 (2)	C28—H28B	0.9700
C8—N2	1.379 (2)	C29—N3	1.452 (2)
C8—C9	1.413 (3)	C29—C30	1.510 (3)
C8—C13	1.417 (3)	C29—H29A	0.9700
C9—C10	1.363 (3)	C29—H29B	0.9700
C9—H9	0.9300	C30—C35	1.366 (3)
C10—C11	1.394 (3)	C30—C31	1.376 (3)
C10—H10	0.9300	C31—C32	1.377 (3)
C11—C12	1.362 (3)	C31—H31	0.9300
C11—H11	0.9300	C32—C33	1.355 (3)
C12—C13	1.402 (3)	C32—H32	0.9300
C12—H12	0.9300	C33—C34	1.357 (4)
C13—N1	1.385 (2)	C33—H33	0.9300
C14—N1	1.301 (2)	C34—C35	1.379 (3)
C14—C15	1.539 (2)	C34—H34	0.9300
C15—N3	1.460 (2)	C35—H35	0.9300
C15—C16	1.564 (2)	C17—O3	1.237 (4)
C16—C17'	1.501 (7)	C17'—O3'	1.237 (9)
C16—C17	1.507 (4)	O2—C26	1.466 (8)
C16—C24	1.557 (2)	C26—C27	1.469 (11)
C16—S1	1.8668 (17)	C26—H26A	0.9700
C16—S1'	1.918 (4)	C26—H26B	0.9700
C18—C23	1.377 (3)	C27—H27A	0.9600
C18—C19	1.379 (3)	C27—H27B	0.9600
C18—C17	1.457 (5)	C27—H27C	0.9600
C18—S1'	1.746 (4)	O2'—C26'	1.463 (5)
C19—C20	1.381 (3)	C26'—C27'	1.471 (9)
C19—H19	0.9300	C26'—H26C	0.9700
C20—C21	1.380 (3)	C26'—H26D	0.9700
C20—H20	0.9300	C27'—H27D	0.9600
C21—C22	1.368 (3)	C27'—H27E	0.9600
C21—H21	0.9300	C27'—H27F	0.9600
			
C2—C1—C6	119.80 (16)	C22—C23—C17'	138.6 (4)
C2—C1—C15	128.32 (15)	C18—C23—S1	117.48 (14)
C6—C1—C15	111.75 (14)	C22—C23—S1	122.10 (15)
C3—C2—C1	118.51 (17)	C17'—C23—S1	16.6 (3)
C3—C2—H2	120.7	C25—C24—C28	113.99 (14)
C1—C2—H2	120.7	C25—C24—C16	115.66 (14)
C4—C3—C2	121.35 (19)	C28—C24—C16	103.37 (13)
C4—C3—H3	119.3	C25—C24—H24	107.8
C2—C3—H3	119.3	C28—C24—H24	107.8
C5—C4—C3	120.65 (18)	C16—C24—H24	107.8
C5—C4—H4	119.7	O1'—C25—O1	15.8 (10)
C3—C4—H4	119.7	O1'—C25—O2	125.9 (10)
C4—C5—C6	118.41 (18)	O1—C25—O2	128.5 (13)
C4—C5—H5	120.8	O1'—C25—O2'	122.2 (6)
C6—C5—H5	120.8	O1—C25—O2'	121.6 (10)
C5—C6—C1	121.24 (17)	O2—C25—O2'	11 (2)
C5—C6—C7	130.07 (16)	O1'—C25—C24	125.7 (4)
C1—C6—C7	108.50 (14)	O1—C25—C24	123.6 (9)
N2—C7—C14	124.02 (16)	O2—C25—C24	107.4 (10)
N2—C7—C6	127.80 (15)	O2'—C25—C24	112.0 (4)
C14—C7—C6	108.17 (14)	N3—C28—C24	105.56 (13)
N2—C8—C9	119.28 (17)	N3—C28—H28A	110.6
N2—C8—C13	121.99 (15)	C24—C28—H28A	110.6
C9—C8—C13	118.73 (17)	N3—C28—H28B	110.6
C10—C9—C8	120.0 (2)	C24—C28—H28B	110.6
C10—C9—H9	120.0	H28A—C28—H28B	108.8
C8—C9—H9	120.0	N3—C29—C30	110.65 (14)
C9—C10—C11	120.96 (19)	N3—C29—H29A	109.5
C9—C10—H10	119.5	C30—C29—H29A	109.5
C11—C10—H10	119.5	N3—C29—H29B	109.5
C12—C11—C10	120.4 (2)	C30—C29—H29B	109.5
C12—C11—H11	119.8	H29A—C29—H29B	108.1
C10—C11—H11	119.8	C35—C30—C31	117.29 (19)
C11—C12—C13	120.4 (2)	C35—C30—C29	121.79 (19)
C11—C12—H12	119.8	C31—C30—C29	120.90 (17)
C13—C12—H12	119.8	C30—C31—C32	121.3 (2)
N1—C13—C12	119.31 (17)	C30—C31—H31	119.3
N1—C13—C8	121.29 (16)	C32—C31—H31	119.3
C12—C13—C8	119.38 (16)	C33—C32—C31	120.3 (2)
N1—C14—C7	123.18 (15)	C33—C32—H32	119.9
N1—C14—C15	126.89 (14)	C31—C32—H32	119.9
C7—C14—C15	109.89 (14)	C32—C33—C34	119.4 (2)
N3—C15—C1	113.41 (13)	C32—C33—H33	120.3
N3—C15—C14	118.30 (13)	C34—C33—H33	120.3
C1—C15—C14	100.59 (12)	C33—C34—C35	120.4 (2)
N3—C15—C16	99.89 (12)	C33—C34—H34	119.8
C1—C15—C16	116.01 (13)	C35—C34—H34	119.8
C14—C15—C16	109.41 (13)	C30—C35—C34	121.3 (2)
C17'—C16—C17	91.1 (4)	C30—C35—H35	119.3
C17'—C16—C24	113.9 (3)	C34—C35—H35	119.3
C17—C16—C24	114.6 (2)	C14—N1—C13	114.93 (14)
C17'—C16—C15	120.7 (3)	C7—N2—C8	114.20 (15)
C17—C16—C15	118.0 (2)	C29—N3—C15	117.32 (13)
C24—C16—C15	99.64 (12)	C29—N3—C28	115.01 (15)
C17'—C16—S1	14.2 (3)	C15—N3—C28	109.42 (13)
C17—C16—S1	105.23 (18)	C23—S1—C16	90.15 (9)
C24—C16—S1	107.81 (10)	O3—C17—C18	124.5 (4)
C15—C16—S1	111.28 (11)	O3—C17—C16	120.5 (4)
C17'—C16—S1'	103.4 (3)	C18—C17—C16	115.0 (3)
C17—C16—S1'	12.4 (2)	C18—S1'—C16	85.91 (18)
C24—C16—S1'	109.57 (17)	O3'—C17'—C23	121.7 (7)
C15—C16—S1'	109.50 (16)	O3'—C17'—C16	117.7 (7)
S1—C16—S1'	117.53 (14)	C23—C17'—C16	120.5 (6)
C23—C18—C19	121.00 (17)	C25—O2—C26	116.1 (16)
C23—C18—C17	109.8 (2)	O2—C26—C27	110.6 (17)
C19—C18—C17	129.2 (2)	O2—C26—H26A	109.5
C23—C18—S1'	126.63 (19)	C27—C26—H26A	109.5
C19—C18—S1'	112.34 (19)	O2—C26—H26B	109.5
C17—C18—S1'	16.89 (18)	C27—C26—H26B	109.5
C18—C19—C20	118.5 (2)	H26A—C26—H26B	108.1
C18—C19—H19	120.8	C25—O2'—C26'	118.5 (6)
C20—C19—H19	120.8	O2'—C26'—C27'	106.5 (7)
C21—C20—C19	120.4 (2)	O2'—C26'—H26C	110.4
C21—C20—H20	119.8	C27'—C26'—H26C	110.4
C19—C20—H20	119.8	O2'—C26'—H26D	110.4
C22—C21—C20	121.4 (2)	C27'—C26'—H26D	110.4
C22—C21—H21	119.3	H26C—C26'—H26D	108.6
C20—C21—H21	119.3	C26'—C27'—H27D	109.5
C21—C22—C23	118.37 (19)	C26'—C27'—H27E	109.5
C21—C22—H22	120.8	H27D—C27'—H27E	109.5
C23—C22—H22	120.8	C26'—C27'—H27F	109.5
C18—C23—C22	120.41 (17)	H27D—C27'—H27F	109.5
C18—C23—C17'	100.9 (3)	H27E—C27'—H27F	109.5
			
C6—C1—C2—C3	1.2 (3)	C28—C24—C25—O2'	−168.1 (7)
C15—C1—C2—C3	176.76 (17)	C16—C24—C25—O2'	72.3 (7)
C1—C2—C3—C4	0.5 (3)	C25—C24—C28—N3	−143.07 (15)
C2—C3—C4—C5	−1.3 (3)	C16—C24—C28—N3	−16.71 (17)
C3—C4—C5—C6	0.4 (3)	N3—C29—C30—C35	132.1 (2)
C4—C5—C6—C1	1.3 (3)	N3—C29—C30—C31	−46.3 (3)
C4—C5—C6—C7	−173.14 (18)	C35—C30—C31—C32	0.1 (3)
C2—C1—C6—C5	−2.1 (3)	C29—C30—C31—C32	178.6 (2)
C15—C1—C6—C5	−178.35 (16)	C30—C31—C32—C33	0.2 (4)
C2—C1—C6—C7	173.39 (15)	C31—C32—C33—C34	−0.3 (4)
C15—C1—C6—C7	−2.86 (19)	C32—C33—C34—C35	−0.1 (4)
C5—C6—C7—N2	−8.4 (3)	C31—C30—C35—C34	−0.5 (4)
C1—C6—C7—N2	176.64 (17)	C29—C30—C35—C34	−178.9 (2)
C5—C6—C7—C14	170.72 (18)	C33—C34—C35—C30	0.5 (4)
C1—C6—C7—C14	−4.24 (19)	C7—C14—N1—C13	−5.0 (2)
N2—C8—C9—C10	177.88 (18)	C15—C14—N1—C13	172.37 (15)
C13—C8—C9—C10	−2.0 (3)	C12—C13—N1—C14	−178.80 (16)
C8—C9—C10—C11	−0.6 (3)	C8—C13—N1—C14	−0.4 (2)
C9—C10—C11—C12	1.8 (3)	C14—C7—N2—C8	−1.6 (2)
C10—C11—C12—C13	−0.5 (3)	C6—C7—N2—C8	177.39 (16)
C11—C12—C13—N1	176.31 (18)	C9—C8—N2—C7	176.33 (16)
C11—C12—C13—C8	−2.1 (3)	C13—C8—N2—C7	−3.8 (2)
N2—C8—C13—N1	5.0 (3)	C30—C29—N3—C15	160.74 (15)
C9—C8—C13—N1	−175.06 (17)	C30—C29—N3—C28	−68.4 (2)
N2—C8—C13—C12	−176.56 (16)	C1—C15—N3—C29	−64.6 (2)
C9—C8—C13—C12	3.3 (3)	C14—C15—N3—C29	52.8 (2)
N2—C7—C14—N1	6.5 (3)	C16—C15—N3—C29	171.26 (15)
C6—C7—C14—N1	−172.68 (15)	C1—C15—N3—C28	161.98 (14)
N2—C7—C14—C15	−171.27 (16)	C14—C15—N3—C28	−80.61 (18)
C6—C7—C14—C15	9.57 (18)	C16—C15—N3—C28	37.89 (16)
C2—C1—C15—N3	−40.5 (2)	C24—C28—N3—C29	−148.29 (15)
C6—C1—C15—N3	135.37 (15)	C24—C28—N3—C15	−13.75 (18)
C2—C1—C15—C14	−167.82 (17)	C18—C23—S1—C16	−9.81 (15)
C6—C1—C15—C14	8.03 (18)	C22—C23—S1—C16	169.01 (16)
C2—C1—C15—C16	74.3 (2)	C17'—C23—S1—C16	−7.5 (10)
C6—C1—C15—C16	−109.83 (16)	C17'—C16—S1—C23	8.5 (12)
N1—C14—C15—N3	47.8 (2)	C17—C16—S1—C23	13.5 (2)
C7—C14—C15—N3	−134.57 (15)	C24—C16—S1—C23	−109.24 (12)
N1—C14—C15—C1	171.82 (16)	C15—C16—S1—C23	142.47 (12)
C7—C14—C15—C1	−10.54 (17)	S1'—C16—S1—C23	15.10 (17)
N1—C14—C15—C16	−65.6 (2)	C23—C18—C17—O3	−172.3 (5)
C7—C14—C15—C16	112.06 (15)	C19—C18—C17—O3	11.1 (7)
N3—C15—C16—C17'	79.5 (4)	S1'—C18—C17—O3	5.5 (7)
C1—C15—C16—C17'	−42.7 (4)	C23—C18—C17—C16	9.5 (4)
C14—C15—C16—C17'	−155.6 (4)	C19—C18—C17—C16	−167.1 (2)
N3—C15—C16—C17	−170.5 (2)	S1'—C18—C17—C16	−172.7 (14)
C1—C15—C16—C17	67.2 (3)	C17'—C16—C17—O3	167.2 (6)
C14—C15—C16—C17	−45.6 (3)	C24—C16—C17—O3	−75.7 (5)
N3—C15—C16—C24	−45.81 (14)	C15—C16—C17—O3	41.2 (6)
C1—C15—C16—C24	−168.07 (13)	S1—C16—C17—O3	166.0 (4)
C14—C15—C16—C24	79.06 (14)	S1'—C16—C17—O3	−7.3 (10)
N3—C15—C16—S1	67.71 (13)	C17'—C16—C17—C18	−14.5 (4)
C1—C15—C16—S1	−54.55 (16)	C24—C16—C17—C18	102.5 (3)
C14—C15—C16—S1	−167.42 (10)	C15—C16—C17—C18	−140.5 (3)
N3—C15—C16—S1'	−160.68 (16)	S1—C16—C17—C18	−15.7 (4)
C1—C15—C16—S1'	77.06 (19)	S1'—C16—C17—C18	171.0 (17)
C14—C15—C16—S1'	−35.81 (19)	C23—C18—S1'—C16	7.8 (3)
C23—C18—C19—C20	−0.8 (3)	C19—C18—S1'—C16	−170.09 (17)
C17—C18—C19—C20	175.5 (3)	C17—C18—S1'—C16	5.2 (10)
S1'—C18—C19—C20	177.3 (2)	C17'—C16—S1'—C18	−12.4 (4)
C18—C19—C20—C21	0.4 (4)	C17—C16—S1'—C18	−6.8 (13)
C19—C20—C21—C22	0.5 (4)	C24—C16—S1'—C18	109.36 (17)
C20—C21—C22—C23	−1.0 (4)	C15—C16—S1'—C18	−142.32 (16)
C19—C18—C23—C22	0.3 (3)	S1—C16—S1'—C18	−14.1 (2)
C17—C18—C23—C22	−176.7 (3)	C18—C23—C17'—O3'	171.1 (7)
S1'—C18—C23—C22	−177.5 (2)	C22—C23—C17'—O3'	−11.3 (10)
C19—C18—C23—C17'	178.5 (3)	S1—C23—C17'—O3'	−6.8 (7)
C17—C18—C23—C17'	1.5 (4)	C18—C23—C17'—C16	−13.0 (6)
S1'—C18—C23—C17'	0.7 (4)	C22—C23—C17'—C16	164.7 (3)
C19—C18—C23—S1	179.12 (16)	S1—C23—C17'—C16	169.1 (15)
C17—C18—C23—S1	2.2 (3)	C17—C16—C17'—O3'	−167.1 (7)
S1'—C18—C23—S1	1.4 (3)	C24—C16—C17'—O3'	75.2 (7)
C21—C22—C23—C18	0.6 (3)	C15—C16—C17'—O3'	−43.2 (8)
C21—C22—C23—C17'	−176.7 (5)	S1—C16—C17'—O3'	8.1 (8)
C21—C22—C23—S1	−178.15 (17)	S1'—C16—C17'—O3'	−165.9 (6)
C17'—C16—C24—C25	33.8 (4)	C17—C16—C17'—C23	16.8 (5)
C17—C16—C24—C25	−69.3 (2)	C24—C16—C17'—C23	−100.9 (5)
C15—C16—C24—C25	163.65 (14)	C15—C16—C17'—C23	140.7 (4)
S1—C16—C24—C25	47.47 (17)	S1—C16—C17'—C23	−168.0 (16)
S1'—C16—C24—C25	−81.5 (2)	S1'—C16—C17'—C23	18.0 (6)
C17'—C16—C24—C28	−91.5 (4)	O1'—C25—O2—C26	−9 (3)
C17—C16—C24—C28	165.4 (2)	O1—C25—O2—C26	11 (4)
C15—C16—C24—C28	38.36 (15)	O2'—C25—O2—C26	65 (7)
S1—C16—C24—C28	−77.82 (14)	C24—C25—O2—C26	−177.7 (19)
S1'—C16—C24—C28	153.18 (16)	C25—O2—C26—C27	−116 (3)
C28—C24—C25—O1'	12.4 (7)	O1'—C25—O2'—C26'	0.9 (16)
C16—C24—C25—O1'	−107.2 (6)	O1—C25—O2'—C26'	19.6 (17)
C28—C24—C25—O1	−6.7 (9)	O2—C25—O2'—C26'	−112 (8)
C16—C24—C25—O1	−126.3 (9)	C24—C25—O2'—C26'	−178.6 (9)
C28—C24—C25—O2	−178.4 (16)	C25—O2'—C26'—C27'	179.5 (13)
C16—C24—C25—O2	61.9 (16)		

### Hydrogen-bond geometry (Å, º)

**Table:**

*D*—H···*A*	*D*—H	H···*A*	*D*···*A*	*D*—H···*A*
C11—H11···O3^i^	0.93	2.38	3.222 (3)	150
C22—H22···N1^ii^	0.93	2.61	3.523 (3)	167

Symmetry codes: (i) −*x*+1, −*y*+1, −*z*+1; (ii) *x*, −*y*+3/2, *z*−1/2.

## References

[bb1] Bruker (2008). *APEX2*, *SADABS* and *SAINT*. Bruker AXS Inc., Madison, Wisconsin, USA.

[bb2] Dandia, A., Singh, R., Bhaskaran, S. & Samant, S. D. (2011). *Green Chem.* **13**, 1852–1859.

[bb4] Farrugia, L. J. (2012). *J. Appl. Cryst.* **45**, 849–854.

[bb5] He, W., Myers, M. R., Hanney, B., Spada, A. P., Bilder, G., Galzcinski, H., Amin, D., Needle, S., Page, K., Jayyosi, Z. & Perrone, H. (2003). *Bioorg. Med. Chem. Lett.* **13**, 3097–3100. 10.1016/s0960-894x(03)00655-3 12941342

[bb6] Kannan, P. S., Lanka, S., Thennarasu, S., Vimala, G. & SubbiahPandi, A. (2013). *Acta Cryst.* E**69**, o854–o855. 10.1107/S1600536813011537 PMC3684937 23795039

[bb7] Pradhan, R., Patra, M., Behera, A. K., Mishra, B. K. & Behera, R. K. (2006). *Tetrahedron*, **62**, 779–828.

[bb8] Raj, A. A., Raghunathan, R., SrideviKumari, M. R. & Raman, N. (2003). *Bioorg. Med. Chem.* **11**, 407–419. 10.1016/s0968-0896(02)00439-x 12517436

[bb9] Saeedi, M., Heravi, M. M., Beheshtiha, Y. S. & Oskooie, H. A. (2010). *Tetrahedron*, **66**, 5345–5348.

[bb10] Sheldrick, G. M. (2008). *Acta Cryst.* A**64**, 112–122. 10.1107/S0108767307043930 18156677

[bb11] Spek, A. L. (2009). *Acta Cryst.* D**65**, 148–155. 10.1107/S090744490804362X PMC2631630 19171970

[bb12] Zarranz, B., Jaso, A., Aldana, I. & Monge, A. (2003). *Bioorg. Med. Chem.* **11**, 2149–2156. 10.1016/s0968-0896(03)00119-6 12713824

