# Supplementary figures and images for: Crystal structure of ethyl (2S,2′R)-1′-benzyl-3-oxo-3H-di­spiro­[1-benzo­thio­phene-2,3′-pyrrolidine-2′,11′′-indeno[1,2-b]quinoxaline]-4′-carboxyl­ate
Source: Acta Crystallogr E Crystallogr Commun. 2015 Feb 21;71(Pt 3):o195–6. doi: 10.1107/S2056989015003187 (PMC4350717; doi:10.1107/S2056989015003187)

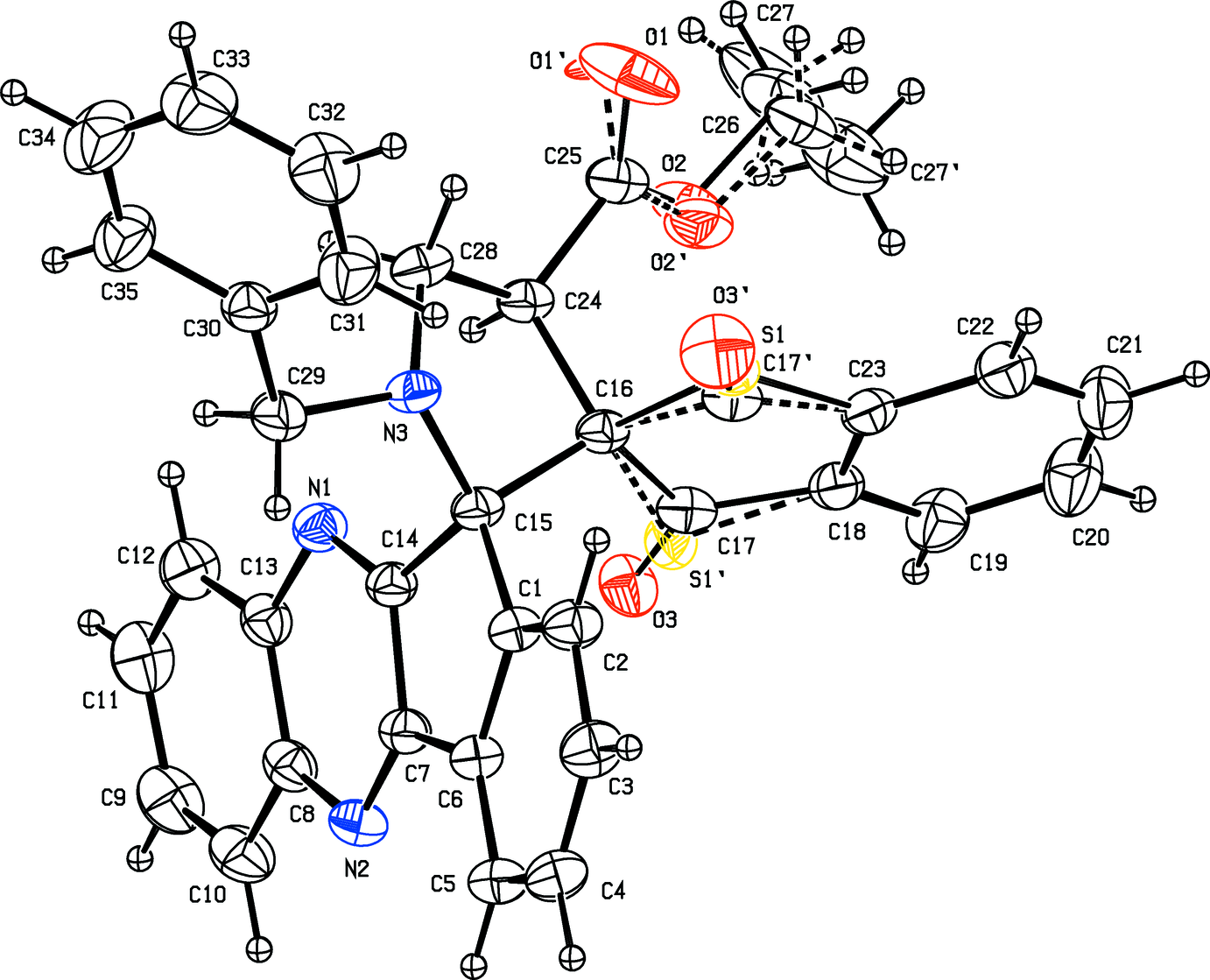

Supplement: Supplementary file 3 [file e-71-0o195-fig1.tif]

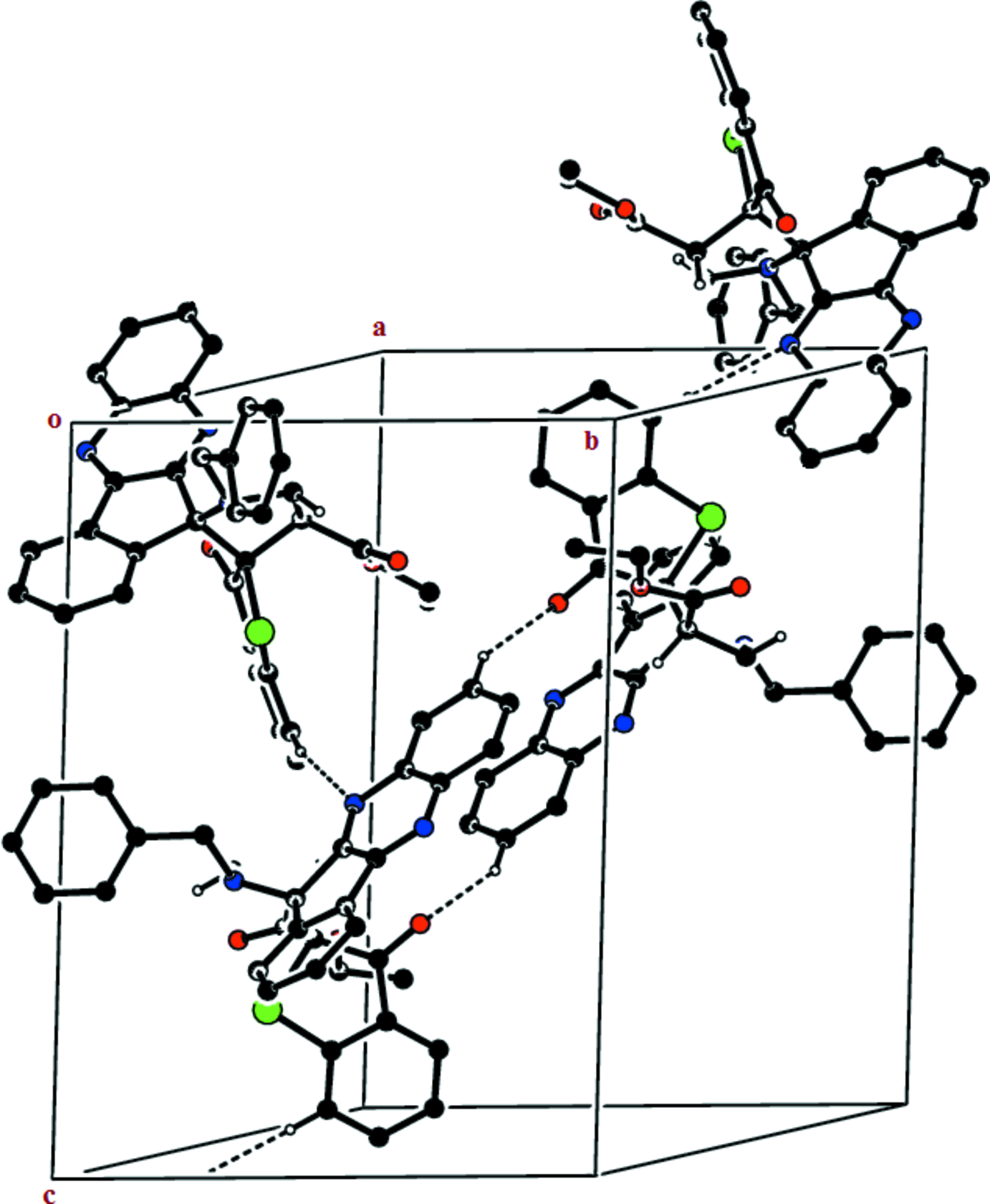

Supplement: Supplementary file 4 [file e-71-0o195-fig2.tif]
